# Supplementary material for: Clinicohematological and molecular analysis of hemoglobin D syndrome and unknown variants in the hemoglobinopathy spectrum of Sindh, Pakistan
Source: PLoS One. 2025 May 15;20(5):e0320354. doi: 10.1371/journal.pone.0320354 (PMC12080823; doi:10.1371/journal.pone.0320354)
Supplement: S1 Table — N: frequency, %: percentage, SD: Standard deviation. (DOCX) [file pone.0320354.s001.docx]

**S1 Table. Clinical Parameters in HbD and HbQ syndromes**

| **Parameters** | **HbD Trait** | **HbD Disease** | **HbDβ Thal** | **HbSD Disease** | **HbDQ Disease** | **HbQ Trait** | **HbQβ Thal** |
| --- | --- | --- | --- | --- | --- | --- | --- |
| **Frequency**  **(N = 110; %)** | 77 (70) | 12 (10.9) | 5 (4.5) | 4 (3.6) | 1 (0.9) | 9 (8.2) | 2 (1.8) |
| **Age (years)**  **(Mean + SD; Range)** | 11+11.2  (0.4-45) | 17+8.6  (2-30) | 4.4+5.6  (0.75-14) | 14.7+15.3  (1.5-34) | 17 | 12.8+10  (2.5-30) | 31+1.4  (30-32) |
| **Age Categories (N; %)** | | | | | | | |
| **<6 months (N = 2)** | 2 (2.6) | 0 (0.0) | 0 (0.0) | 0 (0.0) | 0 (0.0) | 0 (0.0) | 0 (0.0) |
| **6 months – 12 years (N = 61)** | 46 (59.7) | 4 (33.4) | 4 (80) | 2 (50) | 0 (0.0) | 5 (55.5) | 0 (0.0) |
| **>12 years (N = 47)** | 29 (37.6) | 8 (66.6) | 1 (20) | 2 (50) | 1 (100) | 4 (44.5) | 2 (100) |
| **Gender (N; %)** | | | | | | | |
| **Male (N = 62)** | 46 (59.7) | 5 (41.7) | 3 (60) | 4 (100) | 0 (0.0) | 4 (44.4) | 0 (0.0) |
| **Female (N = 48)** | 31 (40.3) | 7 (58.3) | 2 (40) | 0 (0.0) | 1 (100) | 5 (55.6) | 2 (100) |
| **Anemia (N; %)** | | | | | | | |
| **No (N = 19)** | 13 (16.9) | 4 (33.3) | 0 (0.0) | 0 (0.0) | 1 (100) | 1 (11.1) | 0 (0.0) |
| **Yes (N = 91)** | 64 (83.1) | 8 (66.7) | 5 (100) | 4 (100) | 0 (0) | 8 (88.9) | 2 (100) |
| **Mild (N = 18)** | 9 (11.7) | 5 (41.7) | 1 (20) | 0 (0.0) | 0 (0.0) | 1 (11.1) | 2 (100) |
| **Moderate (n = 32)** | 26 (33.8) | 0 (0.0) | 1 (20) | 1 (25) | 0 (0.0) | 4 (44.4) | 0 (0.0) |
| **Severe (N = 41)** | 29 (37.7) | 3 (25) | 3 (60) | 3 (75) | 0 (0.0) | 3 (33.3) | 0 (0.0) |
| **Nutritional deficiency (N; %)** | | | | | | | |
| **Iron deficiency**  **(N = 30)** | 23 (29.9) | 2 (16.7) | 1 (20) | 0 (0.0) | 0 (0.0) | 4 (44.4) | 0 (0.0) |
| **B12 and Folate deficiency (N = 1)** | 1 (1.3) | 0 (0.0) | 0 (0.0) | 0 (0.0) | 0 (0.0) | 0 (0.0) | 0 (0.0) |

N: frequency, %: percentage, SD: Standard deviation
