# Supplementary figures and images for: Clinicohematological and molecular analysis of hemoglobin D syndrome and unknown variants in the hemoglobinopathy spectrum of Sindh, Pakistan
Source: PLoS One. 2025 May 15;20(5):e0320354. doi: 10.1371/journal.pone.0320354 (PMC12080823; doi:10.1371/journal.pone.0320354)

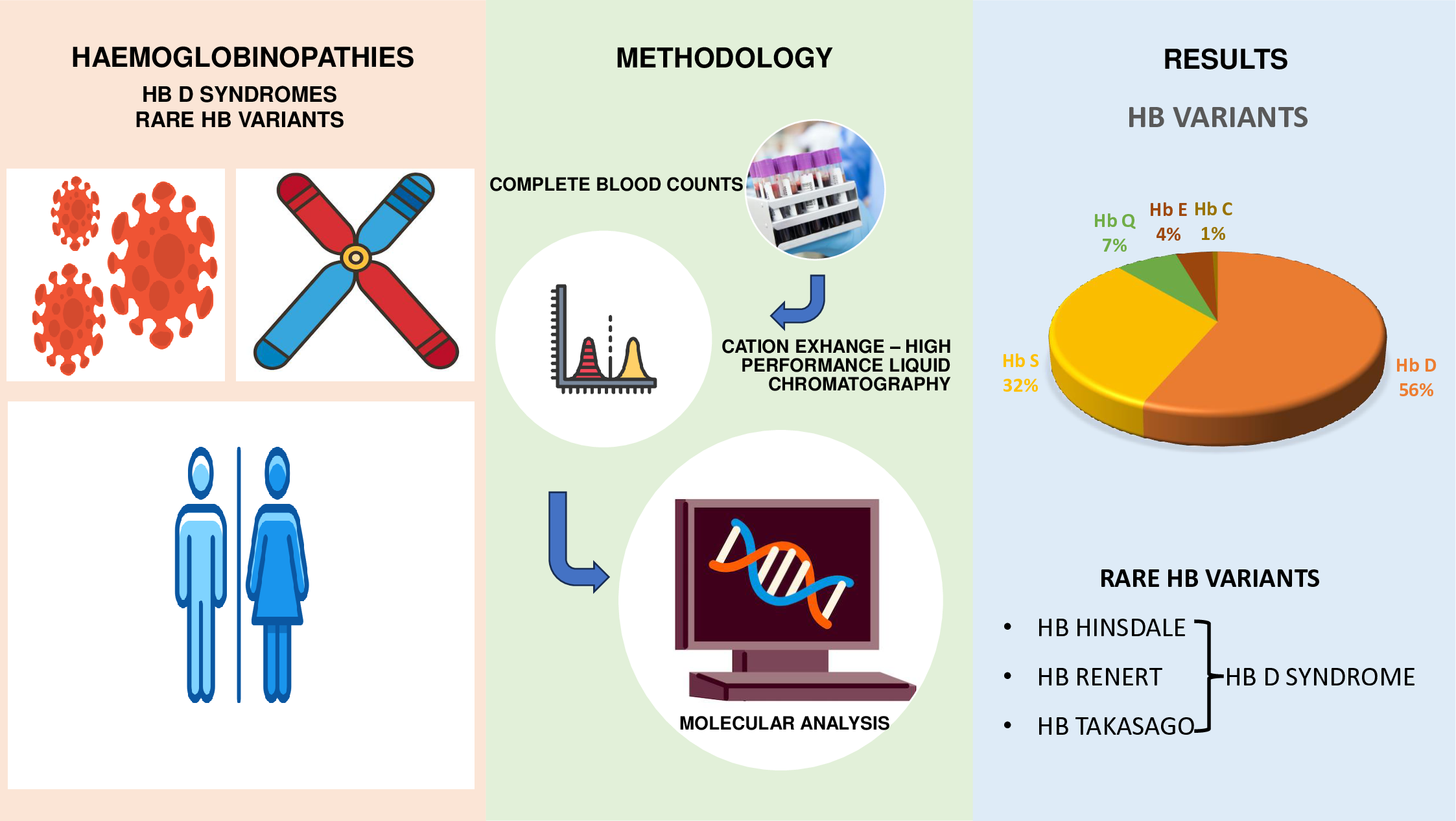

Supplement: S1 — (TIF) [file pone.0320354.s002.tif]
