# Supplementary material for: Clinicohematological and molecular analysis of hemoglobin D syndrome and unknown variants in the hemoglobinopathy spectrum of Sindh, Pakistan
Source: PLoS One. 2025 May 15;20(5):e0320354. doi: 10.1371/journal.pone.0320354 (PMC12080823; doi:10.1371/journal.pone.0320354)
Supplement: Attachment — Submitted filename: comments.docx [file pone.0320354.s003.docx]

The study investigated the prevalence and molecular characteristics of hemoglobin D (HbD) syndrome and other hemoglobin variants in Sindh, Pakistan. It identifies HbD Punjab as the most common variant, followed by sickle cell disorders and HbQ. The research highlights the importance of molecular analysis for accurate diagnosis and suggests integrating HbD detection into screening programs to prevent hemoglobinopathies. The findings emphasize the need for genetic counseling and prenatal diagnosis to manage and reduce the burden of these disorders. The manuscript could benefit from some minor revision points:

1. Authors could provide a graphical abstract to summarizes the work graphically, potentially stimulating researchers to view the published manuscript.
2. There are some areas where sentence structure could be improved for clarity and flow. Consider a thorough revision for grammatical consistency also.
3. More contextual background could be added to justify why this region is a focal point of the study. The mention of Sindh's genetic diversity is insightful, but adding data on the prevalence of hemoglobinopathies in the region (and comparison to other regions in Pakistan) would strengthen the rationale for the study.
4. The images uploaded are blurred and are of low quality, authors should improve the quality of the images.
5. In Table 1, 2, 3 and 4 clearly describe the values in brackets “( )” in caption or below the table.
6. The methodology is well-described, but some subsections could use additional detail. For instance, the **inclusion and exclusion criteria** could be expanded, especially regarding patients with prior blood transfusions (time period of prior transfusion), as this might significantly affect results. Additionally, clarify whether there was any control group or comparative analysis with other populations.
7. Consider providing more statistical depth, especially in comparing various hemoglobinopathies and their prevalence among age and gender groups. This would lend additional robustness to the data and findings.
8. It will be beneficial to include more recent studies, particularly those published within the last 4-5 years, to reflect the current state of research on hemoglobinopathies.
