# Supplementary material for: Clinicohematological and molecular analysis of hemoglobin D syndrome and unknown variants in the hemoglobinopathy spectrum of Sindh, Pakistan
Source: PLoS One. 2025 May 15;20(5):e0320354. doi: 10.1371/journal.pone.0320354 (PMC12080823; doi:10.1371/journal.pone.0320354)
Supplement: Attachment — Submitted filename: Rebuttal letter.docx [file pone.0320354.s005.docx]

**RESPONSE LETTER**

**Academic Editor comments and responses:**

**Comment 1:** Please ensure that your manuscript meets PLOS ONE's style requirements, including those for file naming.

**Response**: Formatting guidelines for Title, author, affiliations and manuscripts were checked and amended accordingly. Updated formatting is highlighted

**Comment 2:** We suggest you thoroughly copyedit your manuscript for language usage, spelling, and grammar. Upon resubmission, please provide the following: A copy of your manuscript showing your changes by either highlighting them or using track changes (uploaded as a *supporting information* file). A clean copy of the edited manuscript (uploaded as the new *manuscript* file)

**Response**: We have thoroughly copyedited manuscript for language usage, spelling, and grammar in revised draft. Both files—a version with tracked changes and a clean copy of the edited manuscript—have been submitted as requested.

**Comment 3:** In the online submission form you indicate that your data is not available for proprietary reasons and have provided a contact point for accessing this data. Please note that your current contact point is a co-author on this manuscript. According to our Data Policy, the contact point must not be an author on the manuscript and must be an institutional contact, ideally not an individual. Please revise your data statement to a non-author institutional point of contact, such as a data access or ethics committee, and send this to us via return email. Please also include contact information for the third party organization, and please include the full citation of where the data can be found.

**Response**: We have corrected the data policy in revised submission.

**Comment 4:** We note that Figure 2 in your submission contain [map/satellite] images which may be copyrighted. We require you to either (1) present written permission from the copyright holder to publish these figures specifically under the CC BY 4.0 license, or (2) remove the figures from your submission: 1. You may seek permission from the original copyright holder of Figure 2 to publish the content specifically under the CC BY 4.0 license. 2. If you are unable to obtain permission from the original copyright holder to publish these figures under the CC BY 4.0 license or if the copyright holder’s requirements are incompatible with the CC BY 4.0 license, please either i) remove the figure or ii) supply a replacement figure that complies with the CC BY 4.0 license. Please check copyright information on all replacement figures and update the figure caption with source information. If applicable, please specify in the figure caption text when a figure is similar but not identical to the original image and is therefore for illustrative purposes only.

**Response:** We were unable to obtain permission from the copyright holder for Fig 2. Therefore, we have removed the original figure and replaced it with a public domain map of Sindh Province, sourced from the USGS National Map Viewer (http://viewer.nationalmap.gov/viewer/). The figure caption has been updated accordingly.

**Reviewers' comments:

Comments to the Author**

**Reviewer #1:** The study investigated the prevalence and molecular characteristics of hemoglobin D (HbD) syndrome and other hemoglobin variants in Sindh, Pakistan. It identifies HbD Punjab as the most common variant, followed by sickle cell disorders and HbQ. The research highlights the importance of molecular analysis for accurate diagnosis and suggests integrating HbD detection into screening programs to prevent hemoglobinopathies. The findings emphasize the need for genetic counseling and prenatal diagnosis to manage and reduce the burden of these disorders. The manuscript could benefit from some minor revision points:

**Comment 1:** Authors could provide a graphical abstract to summarizes the work graphically, potentially stimulating researchers to view the published manuscript.

**Response:** We have provided a graphical abstract with revised manuscript.

**Comment 2:** There are some areas where sentence structure could be improved for clarity and flow. Consider a thorough revision for grammatical consistency also.

**Response:** We have thoroughly revised manuscript for language usage, spelling, and grammar in revised draft.

**Comment 3:** More contextual background could be added to justify why this region is a focal point of the study. The mention of Sindh's genetic diversity is insightful, but adding data on the prevalence of hemoglobinopathies in the region (and comparison to other regions in Pakistan) would strengthen the rationale for the study.

**Response:** We have added a comparison of hemoglobin variant frequencies across provinces in Pakistan in the discussion section (Ref: Israr AS, et al.), to provide additional contextual background.

**Comment 4:** The images uploaded are blurred and are of low quality, authors should improve the quality of the images.

**Response:** We have enhanced the image quality using Apex CoVantage PACE 3.0.2.0, adjusted the image dimensions, and converted the JPG/PDF files to high-resolution TIF files for improved clarity.

**Comment 5:** In Table 1, 2, 3 and 4 clearly describe the values in brackets “( )” in caption or below the table.

**Response:** We have clearly described the values below Tables 1, 2, 3, and 4 as requested.

**Comment 6:** The methodology is well-described, but some subsections could use additional detail. For instance, the inclusion and exclusion criteria could be expanded, especially regarding patients with prior blood transfusions (time period of prior transfusion), as this might significantly affect results. Additionally, clarify whether there was any control group or comparative analysis with other populations.

**Response:** We have further elaborated on the exclusion criteria, specifying that patients with blood transfusions within the last 3 months, known cases of thalassemia, and those undergoing repeat testing were excluded from the study. While no control group was included, we conducted comparative analyses with national and international studies, incorporating local, provincial, and regional statistics.

**Comment 7:** Consider providing more statistical depth, especially in comparing various hemoglobinopathies and their prevalence among age and gender groups. This would lend additional robustness to the data and findings.

**Response:** Thank you for the valuable feedback. We have conducted statistical analyses on the distribution of hemoglobinopathies by age and gender using the chi-square test/Fisher’s exact test, as applicable (Table 1) in the revised manuscript.

**Comment 8:** It will be beneficial to include more recent studies, particularly those published within the last 4-5 years, to reflect the current state of research on hemoglobinopathies.

**Response:** Thank you for the suggestion. We have included recent studies: References: 2, 3, 6-9, 17-32, 35-37, 40, to reflect current research on hemoglobinopathies in our manuscript.

**Reviewer #2:** The manuscript describes a series of variants in the Pakistani population. There are following observations on the manuscript:

**Methods section:
Comment 1.** Complete the HPLC method as CE-HPLC

**Response:** HPLC is replaced by CE-HPLC method in manuscript.

**Comment 2.** Add the manufacturer details for the Capillary sequencing platform. Replace Sangers sequencing by Capillary sequencer.

**Response:** Done.

**Comment 3.** Primers for the ARMS PCR should be added as well and the gel picture can be added as a Supplementary figure.

**Response:** The primers for the ARMS PCR used in this study were based on the methods described by Abraham R. et al. The reference has been added in the revised manuscript

**Results section:**
**Comment 1.** The authors have described 3 Hb variants and 1 beta thal mutation in 2 patients. The diagnosis given in table 5 do not tally with the actual diagnosis. ex. Patient 1 has Dbeta thalassemia and not HbD disease, patient 2 also has HbDbeta thalassemia and not HbD Trait. Patient 3 is compound heterozygous for HbD and HbHinsdale. The patient 4 is a case of compound heterozygous for HbD and HbRenert and not Dbeta thalassemia. Patient 5 also has compound heterozygous for HbD and HbTakasago and not HbDTrait.

**Response:** Thank you for your detailed observations. We acknowledge the inconsistencies in the diagnoses listed in Table 4 (previously Table 5), and we appreciate the corrections provided. We have revised the table to accurately reflect each patient's diagnosis based on both hematological findings and molecular results, as suggested, in the revised manuscript.

**Comment 2:** The above may also be a reason for the severity of anemia observed for patient 4 as HbRenert is highly unstable. Was a test for unstable hemoglobins performed for this patient?

**Response:** Thank you for this insight. We agree that the instability of Hb Renert may contribute to the severity of anemia observed in patient 4. However, we did not perform a specific test for unstable hemoglobins for this patient. We recognize that such testing could provide additional insights into the severity of anemia, and we will consider this in future analyses.

**Comment 3:** The results are unclear about the data on HbD syndromes. Are the authors describing the cases after completion of molecular analysis with the genetic classification or are describing cases after the initial HPLC results.

**Response:** Thank you for your comment. In the results section, we are describing the cases based on the findings after the completion of both the initial CE-HPLC results and subsequent molecular analysis. We aimed to provide a comprehensive overview that includes both the hematological data and genetic confirmation to ensure accurate diagnosis.

**Comment 4:** Severe anemia in many patients remains unexplained in all the conditions.

**Response:** Thank you for your observation. Severe anemia was observed in 41 cases, which can largely be explained by the presence of a compound heterozygous state and nutritional deficiencies in these patients. Specifically, 30 patients had iron deficiency anemia, and 1 patient had B12 and folate deficiency.

**Comment 5:** Cases of compound heterozygous HbD/Q-India have shown mild anemia and not required any transfusions. Hence, the transfusion in the case described needs to be investigated. The same is true of splenomegaly in this patient.

**Response:** While most cases of compound heterozygous HbD/Q-India typically exhibit mild anemia without the need for transfusions, our study identified a case where transfusions were necessary during childhood. The variability in clinical presentation may be attributed to several factors, including the presence of coexisting conditions, such as nutritional deficiencies or infections, that can exacerbate anemia. Our findings suggest that while mild anemia is common, unique circumstances in certain patients can lead to more severe manifestations requiring transfusions, which should be explored on a case-by-case basis.

**Comment 6:** Table 2 can be submitted as a supplementary table.

**Response:** Table 2 is added as a supplementary table in the revised manuscript.

**Comment 7:** The way of writing Hb variants should be as per the HGVS nomenclature throughout the manuscript. HBB:c.420T>G, p.Asn140Lys and so on. The authors seem to switch from one format to the other.

**Response:** The variants have been updated as per the HGVS nomenclature in the revised manuscript.

**Comment 8:** rs35699606 should also be expressed in HGVS nomenclature. It is the Cd8/9 mutation. It should be expressed as c.27dupG in preference to the nomenclature used.

**Response:** The variants have been updated as per the HGVS nomenclature in the revised manuscript.

**Comment 9:** The authors should present the chromatograms of these cases with the Sanger sequencing data. Was the case 4 submitted for any additional analysis such as Capillary electrophoresis? This is an intriguing case.

**Response:** Chromatograms and HPLC results for the rare Hb variants have been added to the article. Case 4 was not submitted for capillary electrophoresis, as this analysis was not part of our methodology. The final diagnosis for this case was confirmed through molecular analysis. While CE was not part of our methodology, we appreciate the potential benefits it could bring and will consider it for future studies.

**Discussion:
Comment 1:** Please use HGVS nomenclature for all the variants.

**Response:** The variants have been updated as per the HGVS nomenclature in the revised manuscript.

**Comment 2:** It requires significant shortening.

**Response:** The discussion section has been shortened in the revised manuscript.
