# Supplementary material for: Clinicohematological and molecular analysis of hemoglobin D syndrome and unknown variants in the hemoglobinopathy spectrum of Sindh, Pakistan
Source: PLoS One. 2025 May 15;20(5):e0320354. doi: 10.1371/journal.pone.0320354 (PMC12080823; doi:10.1371/journal.pone.0320354)
Supplement: Attachment — Submitted filename: Response to Reviewers.docx [file pone.0320354.s006.docx]

**RESPONSE LETTER**

**Reviewer comments and responses:**

Reviewer #3

**Comment 1:** There is the need to improve the presentation of the figures.

**Response**: We have worked and improved the presentation of figures using Apex CoVantage PACE 3.0.2.0.

**Comment 2:** The figures are in bad resolution

**Response**: Thank you for your observation. We have improved resolution of all figures according to PLOS guidelines. Resolution of all figures are now between 300-600 dpi and file size is less than 10 MB.

**Comment 3:** All the methodology subsections should be adequately backed up with a reference

**Response**: Following references have been added in the methodology in the revised manuscript.

12. Chakma S, Das S, Chakma K. Detection of abnormal haemoglobin variants and its characterization among anaemicsby high performance liquid chromatography (HPLC): A prospective study from North India. European Journal of Molecular and Clinical Medicine. 2022 Jan 30;9(3):2552-62.

13. World Health Organization. Guideline on haemoglobin cutoffs to define anaemia in individuals and populations. World Health Organization; 2024 Mar 5.

15. Newton CR, Graham A, Heptinstall LE. Analysis of any point mutation in DNA. The amplification refractory mutation system (ARMS). Nucl Acids Res 1989;17:2503–16.

**Comment 4:** A strong problem statement should be provided in the introduction

**Response**: The problem statement has been added in the introduction of the revised manuscript.

**Comment 5:** The novelty of the work is missing

**Response**: Despite extensive global studies on hemoglobin variants, there remains a significant gap in understanding the prevalence, phenotypic spectrum, and molecular characterization of HbD, HbQ syndromes, and other hemoglobinopathies in Sindh, Pakistan. While β-thalassemia has been extensively studied due to its high carrier prevalence in the region, limited data exist on HbD syndromes and their potential interactions with other hemoglobin variants, such as HbS and HbQ. The findings of the study may contribute to the regional and global understanding of hemoglobinopathies, particularly in identifying unique mutation patterns and genotype-phenotype correlations.

The novelty of this work lies in its comprehensive approach to exploring underreported hemoglobinopathies, its focus on a geographically and genetically diverse population of Sindh, Pakistan and its potential to inform diagnostic accuracy, tailored therapeutic strategies, and public health interventions aimed at reducing the burden of hemoglobinopathies in low-resource settings.

The novelty of the study is mentioned in introduction and discussion section of revised manuscript.
